# The Treasures of Renal Isle: Evaluating a Gamified Module While Enhancing Student Learning

**DOI:** 10.1177/23779608241290683

**Published:** 2024-10-17

**Authors:** Shinu Joy, Kimberly Rumsey, Meredith Ford

**Affiliations:** 1 School of Nursing, 12338 University of Texas Medical Branch at Galveston, Galveston, TX, USA

**Keywords:** undergraduate nursing programs, nursing students, nursing education, gamification, innovation

## Abstract

**Introduction:**

Nursing education continues to evolve with the integration of innovative technologies aimed at enhancing student engagement and understanding of complex medical concepts. Gamification, the application of game elements in nongame contexts, emerges as a promising tool in nursing education.

**Method:**

Faculty members collaborated with instructional designers to develop, *The Treasures of the Renal Isles*, a gamified module for nursing education. Drawing upon their expertise, faculty provided content knowledge and identified key gaming elements, while designers crafted a virtual world and storyline to contextualize the learning experience. The module was implemented within the curriculum, and student feedback was solicited through the Gameful Experience Scale (GAMEX), a validated tool for assessing gamified learning experiences. Additionally, exam scores were analyzed to determine the gamified module's impact on learning outcomes.

**Results:**

Analysis of student responses to the GAMEX tool revealed favorable perceptions of the gamified renal module. Specifically, 77.3% of students believed that the module facilitated their understanding of renal content, indicating its efficacy in knowledge acquisition. Moreover, 71% of students expressed a desire for additional gamified modules, highlighting their engagement and interest in this instructional approach. However, despite positive feedback, no significant differences in test scores were observed postimplementation.

**Conclusion:**

The findings of this project highlight the potential of gamification as an interactive learning method in nursing education. While the gamified renal module received positive feedback from students regarding its effectiveness in facilitating understanding, the lack of significant improvement in test scores warrants further refinement. By incorporating student feedback and revising the module accordingly, nursing educators can utilize gamification to bridge the gap between theoretical knowledge and clinical judgment. The development of additional gamified modules holds promise for enriching the learning experience and preparing nursing students for the complexities of clinical practice.

## Introduction

Nursing schools continue to innovate and implement new technology in educating this generation of nursing students. One of the significant challenges facing nursing faculty today is providing realistic opportunities to implement the clinical judgment model in realistic scenarios and experiences. New graduates struggle to filter essential information in real-life scenarios requiring critical thinking and clinical decision-making (Joy et al., 2023). Gamification in nursing education has emerged as a transformative teaching tool that has revolutionized how students engage with and absorb complex medical concepts. As healthcare education continues to evolve, integrating game elements into the nursing curriculum provides a dynamic, innovative approach to learning (Malicki et al., 2020; Reed, 2020).

Gamification as a teaching strategy uses gaming design elements to actively engage the learner in teaching-learning (Márquez-Hernández et al., 2019). The literature suggests that gamification is an innovative teaching strategy where students actively engage directly with the learning content and manipulate the data for personal understanding. Furthermore, gamification increases information retention and improves critical thinking and clinical decision-making while incorporating concepts from past and current courses to foster nursing excellence (Pront et al., 2018; Reed, 2020).

**Figure 1.**
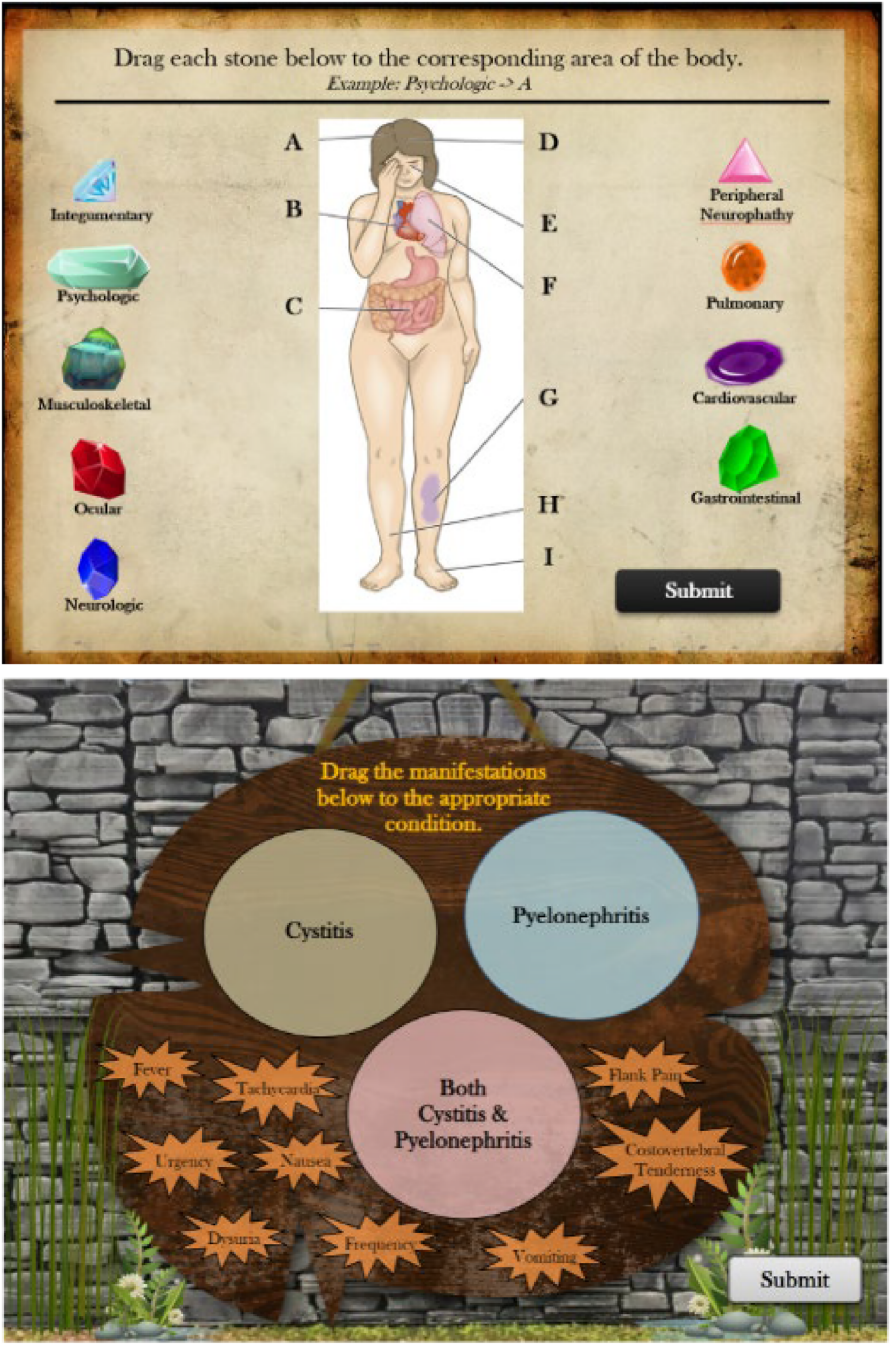
Interactive quizzing methods.

The medical-surgical II course in a prelicensure nursing program analyzed test scores and evaluations from previous semesters revealing that students encountered difficulties in mastering the content specifically within the renal module (Joy et al., 2023). In response, the course faculty opted to enhance student engagement and comprehension by employing gamification strategies for the renal module, presenting the material in a creative and innovative manner. Subsequently, the faculty meticulously designed the module's content to align with gamification principles, incorporating a variety of interactive quiz methods (Figure 1), including terminology checks and case studies, to facilitate deeper learning. The purpose of this quality assessment and improvement project was to assess student satisfaction and the effectiveness of content retention following the implementation of a gamified renal module.

## Methods

The Treasures of the Renal Isles was developed based on the constructivist learning theory to design interactive scenarios to promote autonomous learning and enable feedback mechanisms. In a constructivist framework, learning is an active, student-centered process where knowledge is built upon prior understanding through exploration, problem-solving, and collaboration (Pande & Bharathi, 2020). This engagement helps deepen understanding and retention of nursing concepts.

**Figure 2.**
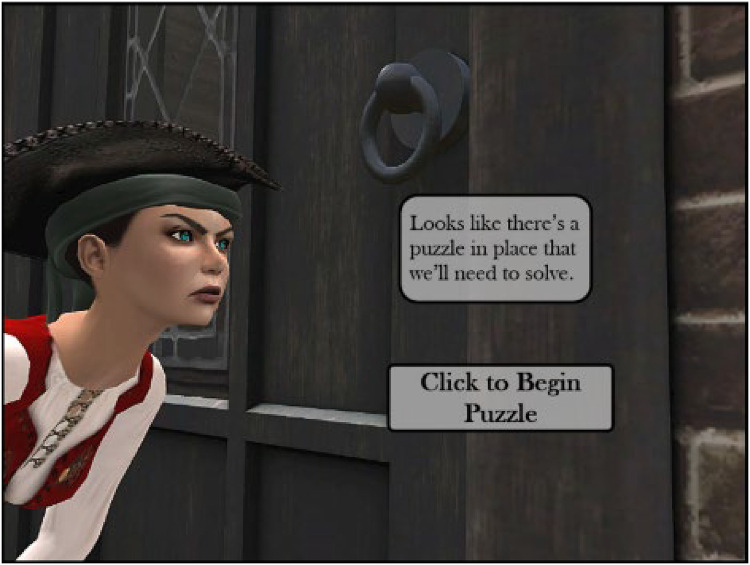
Avatar image.

The educational initiative received approval from the academic health center's Institutional Review Board as a quality assessment and improvement project. The project utilized a convenience sample and was aimed towards second-semester medical-surgical II prelicensure nursing students. There were no ethical issues noted for the project, and informed consent was not required since it was a course activity. Initially, faculty collaborated with instructional designers to identify Articulate as the appropriate software for gamification, selecting a treasure hunt theme to enhance student relatability (Joy et al., 2023). This was followed by drafting the narrative and creating the storyboard. Instructional designers then selected avatars to represent the characters within the storyline (Figure 2). To enhance the module, voice recordings and music were meticulously integrated, ensuring seamless synchronization with the narrative. The faculty's renal content and assessment methods were strategically embedded throughout the storyline to reinforce learning objectives (Joy et al., 2023). Once developed, the module was uploaded to the course's learning management system for students to access. The renal content was taught in a traditional classroom setting and the gamified module was available as supplementary study resource. The gamified module was a voluntary assignment and was piloted for one semester before incorporation into the curriculum. The students received 1.25 points added to their lowest exam grade for completion of the gamified module.

The Gameful Experiences Scale (GAMEX) was used to evaluate the gamified module and student satisfaction. GAMEX, a valid and reliable tool with a Cronbach alpha of > 0.70, consists of six categories: enjoyment, absorption, creative thinking, activation, absence of negative affect, and dominance (Eppmann et al., 2018). In addition, students were asked if they would benefit from other gamified modules. Lastly, feedback was obtained on the overall module to make modifications. The GAMEX survey and the qualitative questions were embedded at the end of the module for students to complete.

## Results

The GAMEX survey was completed by 110 second-semester nursing students. The demographics collected included gender and age. Female participants consisted of 86%, while male participants were 14%. The age range of the participants varied with 38.32% between 16 and 21 years, 38.32% between 22 and 24 years, and 23.36% between 25 and 54 years.

Overall, 77.3% believed the gamified module helped them understand the renal content, and 71% wanted additional gamified modules. Analysis of the GAMEX-specific categories revealed that in the enjoyment category, 73.6% of the students thought the gamified module was fun, and 71.8% liked playing the game. However, only 43.6% would play the game without being asked to. In the absorption category, overall, the students did not feel immersed in the game. Only 31.8% of the students forgot about their immediate surroundings while playing the game. In the creative thinking category, 42.7% felt creative while playing the game, and 41.8% felt adventurous playing the game. The results in the activation category indicated that 47.3% felt activated while playing the gamified module. In the absence of the negative affect category, 71.8% did not feel frustrated while playing the game. Finally, in the dominance category, 40% felt confident playing the game.

A review of the qualitative feedback revealed mostly positive feedback. One student stated, “I really enjoyed completing the module. I am sure if there were other module topics, I would enjoy it just as much while also making learning very interactive and effective.” Another student commented, “I enjoyed playing Treasure on Renal Isle. It was a good way to learn content by playing a game that challenged you as you progressed. It took some of the stress out of studying.” Some students shared their frustration regarding the open-ended case study questions. Students felt having a hint option would be beneficial when answering the free response question. Test scores for the renal content were also analyzed pre- and postimplementation, and test scores did not significantly change for the renal content. The test scores pre-implementation averaged 75.96% and postimplementation was 77.69%.

## Discussion

Gamification is a strategy to allow students to gain new knowledge and enhance their previous knowledge with a nontraditional teaching method (Joy et al., 2023). Quizzing elements of the gamified module included fill in the blanks, select all that apply, and case studies, which resemble next-generation NCLEX (National Council Licensure Examination) questions. Frequent exposure to these styles of questions will better prepare the students to take the licensure exam (Almasloukh et al., 2023). Gamified modules offer a safe and controlled environment for nursing students to refine their understanding of concepts and improve clinical decision-making. Consequently, when transitioning to clinical practice, nurses who have been exposed to gamification may demonstrate increased competence and proficiency in delivering high-quality patient care (Bass et al., 2024). As technology continues to play an increasingly integral role in healthcare delivery, nursing students must possess the necessary technological competencies to navigate digital systems and tools effectively.

Gamified learning environments provide a low-stakes opportunity for nursing students to familiarize themselves with new technologies in a supportive and engaging manner. By incorporating gamification into nursing education programs, educators can help bridge the gap between theoretical knowledge and practical application. Adopting gamification in nursing education can revolutionize the way nurses are trained and prepared for clinical practice.

One area for future investigation includes comparing the effectiveness between traditional instructional methods and gamified approaches across various nursing disciplines to determine which strategies are most effective in enhancing critical thinking, clinical decision-making, and practical skills. Secondly, the integration of advanced technologies such as virtual reality (VR), augmented reality (AR), and artificial intelligence (AI) in gamified nursing education should be explored. As gamification becomes more prevalent, there needs to be a focus on ensuring these interventions are accessible, sustainable, and inclusive to all nursing students. Finally, investigating the long-term effects of gamification will be important in understanding their effectiveness in nursing education.

### Limitations

This project has several limitations. First, the participants were aware they were under assessment to evaluate the gamified module and their satisfaction with the activity, which may have led to bias. The students may have answered the survey questions the way they felt the faculty wanted them to. Another limitation is that students completed these games on their own time so they could be honest about completion and their experience; however, some students may have received help from others. In addition, it was difficult to identify the various ways a student would answer free-response questions, so some acceptable responses could have been marked incorrect. Although the GAMEX survey tool has rarely been used in nursing education (Márquez-Hernández et al., 2019), utilizing this tool will add to the existing literature and demonstrate applicability for use in the nursing education field. According to Márquez-Hernández et al. (2019), the GAMEX tool has high reliability and validity, but the sample it used was based on convenience, which limits the generalizability of the tool's results.

### Implications for Practice

The integration of gamification into nursing education holds several significant implications for nursing practice. Gamification enhances student engagement and motivation. Incorporating points, badges, and immediate feedback, nursing educators can create a more dynamic and interactive learning environment. The heightened engagement can translate into increased retention of information and improved application of knowledge in real-world nursing practice scenarios. Nursing students who are more engaged and motivated in their education are likely to demonstrate greater enthusiasm for lifelong learning, a crucial attribute in the rapidly evolving healthcare landscape. Further research and exploration into the efficacy and scalability of gamification in nursing education are warranted to maximize its impact on nursing practice and patient outcomes.

## Conclusion

The gamified module allowed students to engage in a nontraditional form of learning while retaining the complex renal content. This project specifically evaluated the gamified module and explored the students’ satisfaction with this learning tool. The undergraduate nursing students enjoyed this game-based learning, although the findings did not show significant changes in test scores. According to the GAMEX survey results, the gamification elements provided a positive environment that fostered students’ motivation and desire to learn. These results will allow faculty to modify and enhance the gamified module to ensure continued student success.
